# The Past and the Future

**Published:** 1882-12

**Authors:** 


### HOMŒOPATHIC PHYSICIAN,

A MONTHLY JOURNAL OF MEDICAL SCIENCE.

"If our school ever gives up the strict inductive method of Hahnemann, we are lost, and deserve only to be mentioned as a caricature, in the history of medicine."—CONSTANTINE HERING.

Vol. II. DECEMBER, 1882. No. 12.

### EDITORIAL.

THE PAST AND THE FUTURE.—In the past THE HOMŒOPATHIC PHYSICIAN has been striving to teach true (and useful) Homœopathy; if it has been successful in this endeavor, thanks are due its hard-working contributors. They have supplied us with an abundance of fine material, for which the editor returns his sincere thanks.

During the coming year we hope to continue the good work. Our course will be the same as in the past—to teach *pure* Homœopathy, to illustrate it practically, to bring out the characteristics of old remedies, to introduce new—in short, to do such work as will make us all more efficient in healing the sick. We hope to publish a greater *variety* each month and *shorter* articles. It is our desire to finish, in volume third, the *Cough Symptoms*, the *Notes on Genito-Urinary Therapeutics*, and give some more *Notes from Our Scrap-Book*. Some fine essays from the pen of Dr. P. P. Wells, will be given.

